# A Short Review on the Phase Structures, Oxidation Kinetics, and Mechanical Properties of Complex Ti-Al Alloys

**DOI:** 10.3390/ma14071677

**Published:** 2021-03-29

**Authors:** Hooi Peng Lim, Willey Yun Hsien Liew, Gan Jet Hong Melvin, Zhong-Tao Jiang

**Affiliations:** 1 Faculty of Engineering, Universiti Malaysia Sabah, Jalan UMS, Kota Kinabalu, Sabah 88400, Malaysia; limhooipengjkm@gmail.com (H.P.L.); melvin.gan@ums.edu.my (G.J.H.M.); 2 Surface Analysis and Materials Engineering Research Group, Chemistry and Physics, College of Science, Health, Engineering and Education, Murdoch University, Perth, WA 5150, Australia; Z.Jiang@murdoch.edu.au

**Keywords:** phase structures, oxidation kinetics, oxide scales, Ti-Al alloys, alloying elements

## Abstract

This paper reviews the phase structures and oxidation kinetics of complex Ti-Al alloys at oxidation temperatures in the range of 600–1000 °C. The mass gain and parabolic rate constants of the alloys under isothermal exposure at 100 h (or equivalent to cyclic exposure for 300 cycles) is compared. Of the alloying elements investigated, Si appeared to be the most effective in improving the oxidation resistance of Ti-Al alloys at high temperatures. The effect of alloying elements on the mechanical properties of Ti-Al alloys is also discussed. Significant improvement of the mechanical properties of Ti-Al alloys by element additions has been observed through the formation of new phases, grain refinement, and solid solution strengthening.

## 1. Introduction

In recent years, titanium alloys (Ti-alloys) have often been considered for aircraft, aerospace, shipbuilding, and chemical applications due to their outstanding mechanical performance, and resistance to heat and corrosion. Although many Ti-alloys are relatively stable under ambient environmental conditions, they are known to undergo oxidation at high temperatures. This oxidation is likely to take place during machining, where intense heat is generated [1,2]. Titanium aluminide (TiAl) alloys are often used as structural materials at higher temperatures, due to their high specific strength [3,4], excellent oxidation resistance, high thermal conductivity, and low density [5,6]. The operational temperature of Ti-alloys is usually limited to 600 °C, whereas the maximum temperature for TiAl alloys is 800 °C. Above 600 °C, the formation of non-protective titania (TiO_2_) oxide scale occurs in Ti-alloys. TiO_2_ is loose and porous, allowing oxygen to quickly diffuse inward and to dissolve in the alloys forming a brittle surface layer. TiO_2_ increases in thickness with the inward diffusion of oxygen, which decreases bonding strength at the coating/ substrate interface, thus causing spallation. Furthermore, the formation of TiO_2_ can also lead to lattice distortion due to its brittleness and degrade the mechanical properties of the alloys. Although TiAl alloys show higher oxidation resistance than Ti-alloys, the inconsistent ability of TiAl alloys to form protective alumina (Al_2_O_3_) oxide scale under extended periods of oxidation process has limited their applications.

One feasible method to improve the oxidation behaviour of Ti-alloys and TiAl alloys is the alloying modification process. Adding aluminium (Al) element to Ti-alloys has been found to form a protective layer of Al_2_O_3_ which had a slower oxide growth than TiO_2_, excellent bonding force, and good coating/substrate adhesion at high temperatures [7]. The resistance of Ti-Al alloys to the oxidising environment was increased to temperatures above 800 °C, and the higher content of Al (>60 wt.%) showed excellent oxidation protection with the formation of dense and void-free Al_2_O_3._ The Al_2_O_3_ acted as an oxygen-diffusion barrier that protected the substrate. This diffusion barrier was able to inhibit the inward diffusion of oxygen and the outward diffusion of Ti, hence improving the oxidation resistance of the alloys at high temperature. 

Simultaneously adding alloying elements into Ti-alloys and TiAl alloys, in addition to Al, has also been found to increase their high-temperature oxidation resistance. The addition of alloying elements can suppress the growth of TiO_2_ through the formation of protective oxide scales such as Al_2_O_3_, Cr_2_O_3_, and SiO_2_. More recently, the effect of alloying elements in Ti-Al alloys has been studied. Ti-Al alloys with alloy additions show a smaller difference in thermal expansion coefficient than Ti-alloys and TiAl alloys, due to their similar composition and superior compatibility between alloys and substrate. This characteristic can suppress interdiffusion at the coating/ substrate interface and reduce the spallation and cracking on the surface coating as the oxidation progresses.

Several previous reviews have reported the high-temperature oxidation behaviour of Ti-alloys and TiAl alloys based on thermodynamics, kinetics analysis, the surface modification techniques used [8,9], and the influence of alloying additions to improve oxidation resistance [10]. However, the oxidation behaviour of Ti-Al alloys with alloying additions at high temperatures and the effect of additional elements on the phase formed which affect the mechanical properties are still unclear. Therefore, this paper aims to review the oxidation behaviour of Ti-Al alloys based on their phase structures, oxidation kinetics, and the effect of alloying additions on their mechanical properties. The performance of the Ti-Al alloys is summarised, and information on the deposition method, nominal composition of the alloys, the exposure condition (temperature and oxidation conditions), mass gain, and the parabolic rate constant (under isothermal exposure at 100 h or equivalent to cyclic exposure for 300 cycles) using a reference of 1 mg/cm^2^ mass gain, is provided. The selected exposure condition is close to the phase transformation temperature of the alloys.

## 2. Review Methods

To compare the phase structures and the oxidation kinetics of Ti-alloys and TiAl alloys, experimental studies involving various alloying additions with oxidation exposures in the range of 600 to 1000 °C were reviewed. However, studies on the oxidation behaviour using a modelling method were excluded. The oxidation behaviour of Ti-Al alloys was compared based on their phase structures and oxidation kinetics and also by the effects on their mechanical properties. The exposure condition under isothermal exposure at 100 h (or equivalent to cyclic exposure for 300 cycles) was used to compare the mass gain (mg/cm^2^) and parabolic rate constant (g^2^∙cm^−4^∙s^−1^) of the alloys.

**Figure 1:**
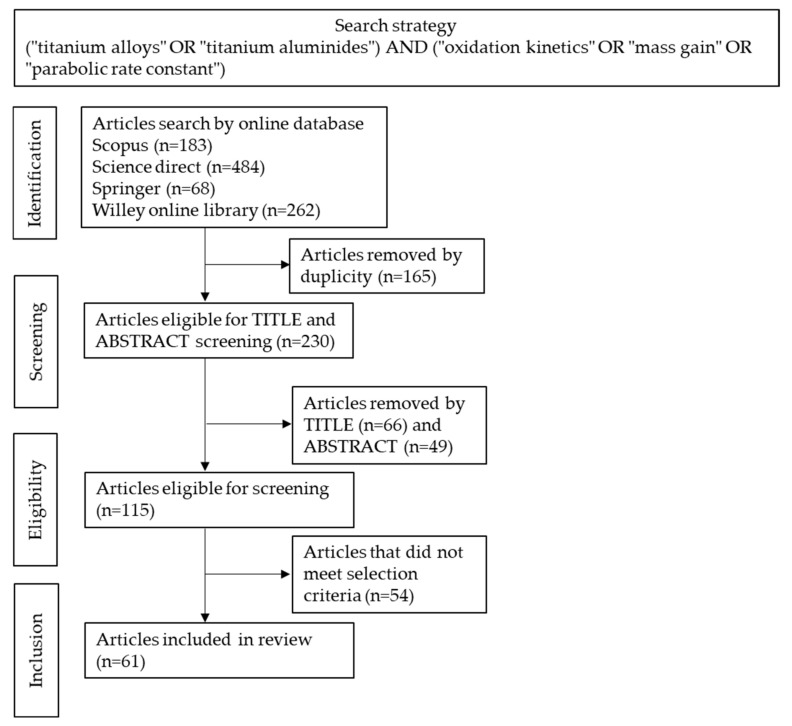
Search strategy used to identify eligible articles for review.

Relevant articles were obtained by search using the Scopus, Science Direct, Springer and Wiley Online Library online databases. The keywords used were: ((“titanium alloys” OR “titanium aluminides”) AND (“oxidation kinetics” OR “mass gain” OR “parabolic rate constant”)). The search was limited to English language articles published from 2000 to 2020. The articles returned by the search were analysed and duplicate articles or articles that did not address Ti-alloys and TiAl alloys were excluded. Articles addressing Ti-alloys and TiAl alloys without alloying addition and without providing the mass gain or parabolic rate constant were only used to support the discussion within the present review. In total, 61 articles meeting these criteria were identified (Figure 1). The other references cited in the present review were used to support the discussion.

## 3. High-Temperature Oxidation of Metallic Materials

In general, an increased oxidation temperature will result in the degradation of metallic materials due to increased diffusion and oxidation rate. When metallic materials are subjected to high temperatures in air, oxidation occurs, leading to the formation of oxide scales. In addition to high-temperature exposure, the crystal structure of the metals themselves influences the oxidation rate of metals [11]. In this section, the high-temperature oxidation of nickel (Ni), zinc (Zn), and iron (Fe) is discussed based on the variation of the parabolic rate constant.

The oxide scales formed for Fe above 570 °C, consisted of wustite phase (FeO), magnetite phase (Fe_3_O_4_), and haematite phase (Fe_2_O_3_). The wustite phase, FeO, was found to form adjacent to the metal due to its high mobility of cations and electrons via the surface vacancies. The outward diffusion of Fe ions was reduced at the wustite-magnetite and magnetite-haematite interfaces. Therefore, the wustite layer was thicker than the magnetite and haematite layers. The variation of the parabolic rate constant was difficult to determine due to the extremely thin haematite layer (approximately 1% of the entire metal-oxide thickness) next to the gas phase, despite the increasing oxygen pressure. Thus, it could be concluded that the parabolic rate constant was unaffected by oxygen pressure at temperature above 570 °C. In contrast, only a two-layer oxide of Fe_3_O_4_ and Fe_2_O_3_ was formed when the Fe oxidised below 570 °C. The rate of scaling at temperature below 570 °C was relatively low due to the absence of the fast-growing FeO. 

Oxidation of Ni formed a single layer oxide of NiO within the temperature range of 500 to 1300 °C [12]. The oxidation and surface kinetics showed notable variation of the parabolic rate constant within three distinct ranges of oxidation temperature. During oxidation at 500 to 600 °C, the oxidation kinetics of NiO was found to follow the parabolic law. In the range of 700 to 1000 °C, two orders of oxidation behaviour were observed. First, the oxidation rate increased rapidly but then decreased to become parabolic; and second, the oxidation rate did not follow a parabolic law and decreased rapidly with time. Above 1100 °C, the oxidation kinetics of NiO again obeyed the parabolic law. The parabolic rate constant $k_{p}$ increased with oxygen pressure as a function of oxidation temperature, suggesting that the oxidation rate of NiO was dependent on oxygen pressure and oxidation temperature. 

Zn also formed a single layer oxide of ZnO upon oxidation. In contrast to NiO, the parabolic rate constant for ZnO was found to be independent of the oxygen pressure due to a minimal concentration difference between interstitial Zn ions and the oxide scale. The parabolic rate constants of ZnO oxidised at 390 °C were 7.2 × 10^−9^ and 7.5 × 10^−9^ g^2^∙cm^−4^∙h^−1^ under oxygen pressures of 1 and 0.022 atm, respectively, indicating a minimal change in the rate constant [13]. 

The thermodynamic stability of different oxides can be compared using an Ellingham diagram, which plots the standard Gibbs free energy (ΔG°) of formation versus temperature for the oxides. The lower position line of the oxides in the diagram indicates oxides with greater stability. The higher the negative Gibbs free energy of oxide dissolution, the lower the equilibrium dissolution of impurity content in the alloys, and thus the higher the thermodynamic stability of the oxides. Cui et al. [14] found that Y_2_O_3_ had the highest thermodynamic stability compared to CaO, MgO, and Al_2_O_3_ in molten Ti and Ti alloys due to the highest Gibbs free energy of formation for oxides (approximately −1897.862 + 281.96*T* kJ/mol, where *T* is the melt temperature). However, an increase in melt temperature was able to increase the chemical reactivity of the alloys which changed their composition and consequently decreased the negative Gibbs free energy. It is known that TiO_2_ and Al_2_O_3_ have similar thermodynamic stability due to their similar Gibbs free energy of formation for oxides. However, TiO_2_ is more stable and tends to grow faster than Al_2_O_3_ due to the strong disorder present in its crystal lattice. The thermodynamic stability of Al_2_O_3_ can be increased by increasing Al content, which also intensifies the activity of Al [15]. Al_2_O_3_ is more stable with an Al content greater than 50 at.%, whereas below this concentration, the thermodynamic stability of TiO_2_ is higher [16].

High-temperature oxidation of metallic materials produces oxides such as protective and non-protective oxide. The protective oxide reduces oxygen diffusion to the coating surface by forming a continuous structure and a well-adhered oxide layer, whereas the non-protective oxide is permeable to oxygen diffusion due to its porous and loose oxide layer. Table 1 shows the Pilling-Bedworth ratio (R_PB_) of metallic oxides and was constructed according to previous studies on the oxidation of metals at high temperature. The R_PB_ value is the index ratio between the volume of oxide and oxidised metal, and it can be used to predict the protection properties of the oxides. The oxide scale is protective when R_PB_ is in the range of 1–2. However, the oxide scale is not sufficient to protect the metal from further oxidation when R_PB_ is less than 1. When R_PB_ exceeds 2, the oxide scale tends to spall off and crack due to the increased stresses in the oxide layer. Al_2_O_3_ (with R_PB_ of 1.28) is expected to have better protection properties against further oxidation at high temperature than TiO_2_ (with R_PB_ of 1.78). While Cr_2_O_3_ and SiO_2_ possess R_PB_ of 2.07 and 2.15, respectively, which are higher than 2, indicating non-protective properties. However, these oxides exhibit excellent oxidation resistance practically based on several previous studies.

In contrast to the high-temperature oxidation of metals, the oxidation of alloys is more complicated due to the formation of oxides, which may dissolve into each other or produce a new phase. The following section discusses the oxidation behaviour of Ti-Al alloys when other alloying elements are added.

## 4. Oxidation Behaviour of Ti-Al Alloys

Oxidation of Ti-alloys often results in the formation of TiO_2,_ which is unfavourable as it is porous and has a high tendency to crack, thereby accelerating the oxidation process. In contrast, the formation of protective oxide scales such as Al_2_O_3_, Cr_2_O_3_, and SiO_2_ in Ti-Al alloys with alloy additions can enhance high-temperature oxidation resistance due to their excellent thermal stability. Tang et al. [19] found that Ti-Al-Cr coatings exhibited excellent isothermal and cyclic oxidation resistance at 800–1000 °C due to the adherent formation of Al_2_O_3_. The coatings were also thermally compatible with the substrate. Wei et al. [20] reported that the formation of Cr_2_O_3_ increased the oxidation resistance of the chromising coatings for temperatures up to 850 °C.

Rare earth elements such as La, Y, and Gd were added to TiAl alloys to investigate their effect on oxidation behaviour in high-temperature oxidising environments. Izumi et al. [21] studied the oxidation behaviour of sulfidation processed TiAl alloys with various alloying elements such as Ta and La at 900 °C in air. The TiAlTa formed from the reaction between Ta-Al and TiAl_3_ was found to provide beneficial protection from further oxidation. In contrast, the formation of La-Al layer on TiAl-La by sulfidation process did not contribute to increased oxidation resistance. Panin et al. [22] reported that the additional Gd could refine the microstructure of Zr- and Cr-containing TiAl alloys, and thus decrease the grain size. Additionally, nano-particles of Gd_2_O_3_ phases were identified, which increased plastic elongation without affecting the strength of the alloys. It can therefore be concluded that the addition of rare earth elements into TiAl alloys is able to improve oxidation resistance due to the excellent stability of the oxide scales formed at high temperature. Rare earth elements with high melting temperatures can stabilise the grain structure, which also contributes to the improved mechanical properties of the alloys.

Although the deposition of coatings such as Al_2_O_3_, SiO_2_, and Cr_2_O_3_ by the sol-gel method [23,24,25] is an alternative method to form oxide scales as the diffusion barrier for the substrate, the effectiveness of such coatings requires further investigation in comparison to thermally-grown oxide. These sol-gel coatings usually crack and spall during long-term oxidation due to their poor adhesion to the substrates. At high oxidation temperatures, the alloyed coating produced by the thermal diffusion process can effectively inhibit the diffusion of oxygen and metal ions due to the excellent metallurgical bonding at the coating/ substrate interface, compared to the former deposition method. 

### 4.1. TiO_2_ Oxide Scale

**Figure 2:**
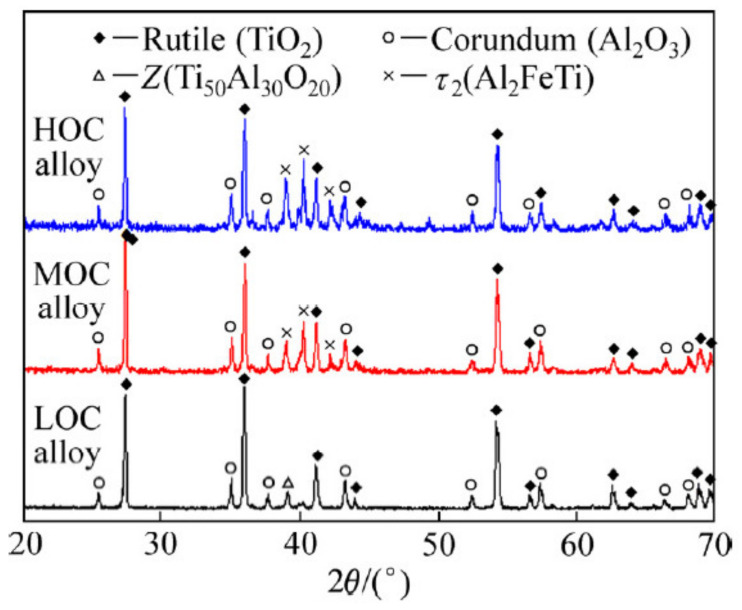
XRD patterns of oxide scales of oxidised low oxygen content (LOC) alloy, medium oxygen content (MOC) alloy, and high oxygen content (HOC) alloy. Reprinted from Transactions of Nonferrous Metals Society of China, 29, Kun Zhao, Si-hui Ouyang, Yong Liu, Bin Liu, Xiao-peng Liang, Hui-zhong Li, Yu Wang, Isothermal oxidation behaviour of TiAl intermetallics with different oxygen contents, 526–533, Copyright (2019), with permission from Elsevier [27].

**Figure 3:**
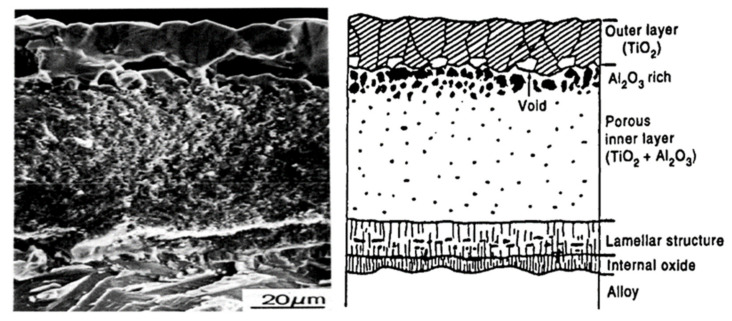
SEM micrograph of TiAl specimen oxidised at 1300 K for 100 ks in oxygen, showing several large voids near the interface between the TiO_2_ outer layer and the TiO_2_ + Al_2_O_3_ interlayer. Reprinted from Intermetallics, 4, Shigeji Taniguchi, Toshio Shibata, Influence of additional elements on the oxidation behaviour of TiAl, S85–S93, Copyright (1996), with permission from Elsevier [10].

Reddy et al. [26] found that a mixture of TiO_2_ and Al_2_O_3_ on Ti-48.6Al alloy oxidised at 850–1000 °C in pure oxygen. The TiO_2_ was loose and porous, which provided a fast diffusion path for oxygen. When the oxidation progressed up to 1000 °C, TiO_2_ overgrew into the oxide scales due to the inward diffusion of oxygen and outward diffusion of Ti. The resulting oxide scales consisted of a TiO_2_ outer layer, on top of a TiO_2_ + Al_2_O_3_ interlayer, with an Al_2_O_3_ enriched layer at the bottom, due to the higher diffusivity of Ti over oxygen. Zhao et al. [27] found that the high oxygen content in TiAl alloy promoted internal oxidation to form interlayer of TiO_2_ and Al_2_O_3_ oxides, and the Al_2_O_3_ enriched layer. The thickness of these oxides increased with dissolved oxygen content. The oxidised products were the predominant phase of TiO_2,_ and the minor phase of Al_2_O_3_, as indicated by the XRD diffraction peak (Figure 2). With increasing oxidation temperature and time, the proportion of TiO_2_ increased and cracks formed near the interface between the TiO_2_ outer layer and the TiO_2_ + Al_2_O_3_ interlayer (Figure 3) [10,28]. In contrast, the formation of Al_2_O_3_ consisted of dense oxide grains, providing better protection against further oxidation by retarding the interdiffusion between oxygen and Ti.

Nevertheless, internal stress was generated when TiO_2_ continued to grow and become thicker due to the increased volume between the oxide and the reacting alloy. The TiO_2_ started to crack and spalled off when the increasing internal stress exceeded the bonding strength at the coating/substrate interface. Dai et al. [29] attributed the cracks formed in the Ti-Al alloys to the difference in the thermal expansion coefficients between the coating and the substrate, which induced significant thermal stress. To retard the growth of the TiO_2_, alloying modification of Ti-alloys and TiAl alloys was developed. The additional alloying elements were required to possess higher valency than Ti^4+^ in order to reduce the concentration and mobility of cracks in TiO_2_. Cations of valence less than Ti^4+^ would accelerate the oxidation rate and promote the diffusion of Ti^4+^ in TiO_2_, resulting in severe spallation and cracks. Therefore, the substitution of Ti^4+^ with high cation valence elements such as Mo^6+^, W^6+^, and Nb^5+^ reduced the oxygen vacancies and effectively prevented the growth rate of TiO_2_. In contrast, the addition of Cr^3+^ increased both the oxygen vacancies and the growth rate of TiO_2_ [30]. However, the detrimental effect of Cr addition on the oxidation resistance could be reduced by increasing the Cr content to more than 8 at.% [31,32]. Higher Cr content promoted the formation of an adherent Al_2_O_3_, thus significantly increasing oxidation resistance.

### 4.2. Al_2_O_3_ Oxide Scale

In general, the oxide scales formed in TiAl alloys above 600 °C are comprised of Al_2_O_3_ and TiO_2_. Unlike TiO_2_, Al_2_O_3_ is compact and adheres well to the substrate; it is a protective oxide scale and exhibits a slower growth rate than TiO_2_ at temperatures up to 1200 °C [33]. The behaviour of Al in Ti-alloys and TiAl alloys during the oxidation process must be investigated in order to understand the oxidation mechanism of the system to prevent high-temperature oxidation. According to the Wagner theory [34], the diffusivity of Al in the alloys must be increased to form protective Al_2_O_3_. This can be achieved by increasing the Al content. Higher Al content increases the proportion of Al_2_O_3_ relative to TiO_2_ because most of the Al diffuses towards the near-surface region during oxidation. 

Since the free energy systems for the formation of Al_2_O_3_ and TiO_2_ are highly similar [35], Al_2_O_3_ and TiO_2_ form simultaneously in the initial oxidation state. However, the strong affinity of Al to oxygen and the higher negative Gibbs free energy of formation for Al_2_O_3_ (ΔG°=−954 kJ/mol) drives the Al ions towards the interface to form more Al_2_O_3_, as compared to TiO_2_ (ΔG°=−944 kJ/mol)[36]. The smaller Al ions diffuse outward towards the surface to form Al_2_O_3_, which serves as a barrier against oxygen for further oxidation. The diffusion process of oxygen has been found to correspond to the oxidation rate of the oxide scales: the higher the diffusivity of oxygen, the higher the oxidation rate of the oxide scales. The oxidation rate can be measured by the oxidation kinetics mass gain of the coatings. Becker et al. [37] found that Al_2_O_3_ dissolved and re-precipitated in the TiO_2_ during oxidation, which accelerated the oxidation rate in Ti-Al alloys. 

**Figure 4:**
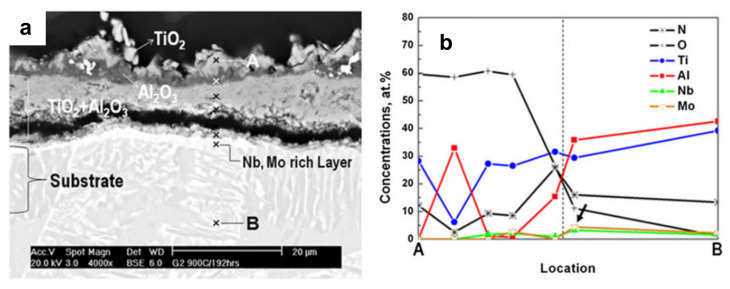
Element distribution on (**a**) the cross-section of oxide scales formed on isothermal exposed TiAl-2Nb-2Mo (G2) alloy at 900 °C for up to 192 h and (**b**) dashed lines represent the location of interface between substrate and oxide layer. Reprinted from Intermetallics, 19, DJ Kim, DY Seo, H Saari, T Sawatzky, Y-W Kim, Isothermal oxidation behavior of powder metallurgy beta gamma TiAl–2Nb–2Mo alloy, 1509-1516, Copyright (2011), with permission from Elsevier [38].

The oxide structures of TiAl-2Nb-2Mo alloy consisted of TiO_2_, Al_2_O_3_, and TiO_2_ + Al_2_O_3_ (Figure 4) [38]. TiO_2_ on the surface was formed by the outward diffusion of Ti, whereas the intermediate Al_2_O_3_ and the inner TiO_2_ + Al_2_O_3_ grew by simultaneous inward diffusion of oxygen and outward diffusion of Al, respectively [38]. Both TiO_2_ and Al_2_O_3_ continued to grow with increasing oxidation temperature and time. The progressive dissolution of oxygen in the Ti-Al alloys increased the mass gain of the oxidised scales in the air. Hence, the diffusion of Ti and Al governed the growth rate of the oxide scales. 

## 5. High-temperature Oxidation Kinetics of Ti-Al Alloys

**Figure 5:**
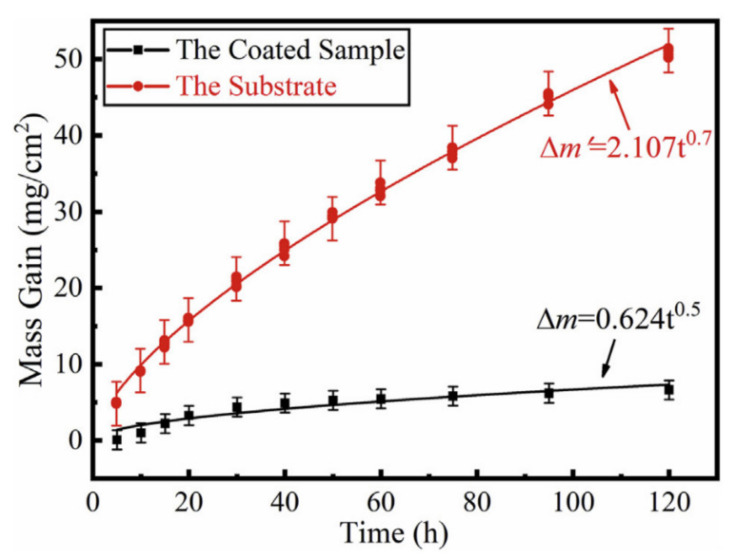
The oxidation kinetic curves (mass gain versus time) of the Ti-Al-Si coating and the substrate of Ti-6Al-4V alloy at 800 °C in air. Reprinted from Journal of Alloys and Compounds, 830, Xiaoyuan Hu, Faguo Li, Dongming Shi, Yu Xie, Zhi Li, Fucheng Yin, A design of self-generated Ti–Al–Si gradient coatings on Ti–6Al–4V alloy based on silicon concentration gradient, 154670, Copyright (2020), with permission from Elsevier [39].

The oxidation kinetics of Ti-Al alloys at high temperatures is usually dominated by the reaction, and the diffusion rates of oxygen and the alloying elements. The higher the diffusivity of metal ions, the faster the oxidation rate. The oxidation kinetics of the alloys ΔW is defined as:

(1) $$\Delta W^{n}=k_{p}t$$

where n is the oxidation reaction index, $k_{p}$ is the parabolic oxidation reaction rate constant, and t is the oxidation time. When the oxidation reaction indices of the alloys are high, and the alloys oxidise at a constant temperature for different oxidation times, higher oxidation rates are found. Mass gain is used to measure the formation of an oxidation barrier. Alloys with high oxidation rates show inferior oxidation resistance with significant mass gain and spallation. Most of the oxidation kinetics curves of Ti-Al alloys follow the parabolic rate curve (Figure 5) [39]. The $k_{p}$ can be determined by the plot of $\Delta W^{2}$ versus t, using a linear regression method where n = 2 for a parabolic relationship. Higher $k_{p}$ values indicate a higher oxidation rate. A decrease in the $k_{p}$ has been observed for Ti-Al alloys upon alloying modification [40]. Alloying elements decreased the oxidation rate of Ti-Al alloys, which showed a variation of oxidation kinetics with respect to their alloy compositions.

### 5.1. Alloying Modification of Ti-Al Alloys

An effective method of improving the high-temperature oxidation resistance of Ti-Al alloys is to modify the alloying composition. The structure of oxide scales influences their high-temperature oxidation resistance and various elements can be added to Ti-Al alloys to enhance the structural performance of their oxide scales. Protective Al_2_O_3_ usually exhibits excellent high-temperature oxidation resistance. At high temperatures, high Al content increases the proportion of Al_2_O_3_ due to the high diffusivity (D_Al_) of Al, thus enhancing the oxidation resistance of Ti-Al alloys. High Al content can be oxidised into Al_2_O_3_ in order to retard the Ti and oxygen diffusion effectively. Other alloying elements such as Si, Nb, Cr, Mo, Co, Cu, Zr, B, La, Fe, Ni, and Ta can also modify the structure of the oxide scales and therefore increase the high-temperature oxidation resistance of Ti-Al alloys. The structural performance of oxide scales includes the promotion of Al_2_O_3_, the formation of the oxygen-diffusion barrier at the coating/substrate interface, and the effect of their chemical composition on oxidation resistance. Studies have shown that oxidation kinetics grows steadily when Ti-Al alloys are oxidised and that as the reaction progressed by increasing temperature, the oxide scales grew continuously, owing to the interdiffusion of oxygen and the metal ions. 

Table 2 summarises the high-temperature exposure, nominal composition, mass gain, and parabolic rate constant for Ti-Al alloy coatings and their uncoated substrates developed by various deposition techniques. The oxidation kinetics of Ti-Al alloy coatings with alloying additions was found to be significantly lower than that of uncoated substrates. This trend in oxidation kinetics obeyed the parabolic law and increased with temperature and time, given in mass gain (mg/cm^2^) and the parabolic rate constant (g^2^∙cm^−4^∙s^−1^). Table 3 shows the same information for bulk Ti-Al alloys prepared by different methods. Alloying additions decreased the mass gain and parabolic rate constant of bulk Ti-Al alloys. Excessive amounts of alloying elements resulted in the formation of the brittle phase and thus deterioration of mechanical properties. To study the performance of high-temperature oxidation resistance of the Ti-Al alloys, the oxidation kinetics and phase structures of the alloys are reviewed at oxidation temperatures in the respective ranges of 600–800 °C and 800–1000 °C. 

### 5.2. Phase Structures and Oxidation Kinetics of Ti-Al Alloys

#### 5.2.1. Oxidation at 600–800 °C

In this section, the phase structures and oxidation kinetics of Ti-Al alloys oxidised upon isothermal and cyclic oxidation at 600–800 °C are reviewed. Ebach-Stahl et al. [41] found that Ti-49.4Al-19.5Cr-0.6Y coated on Ti-6Al-2Sn-4Zr-2Mo and Ti-5Al-2Sn-2Zr-4Mo-4Cr alloys had better oxidation protection than uncoated alloys at 600 and 700 °C under cyclic oxidation in air. The as-deposited thickness of the Ti-49.4Al-19.5Cr-0.6Y coatings was 10 μm. At 600 °C, the Ti-49.4Al-19.5Cr-0.6Y coated alloys consisted of Ti(Cr,Al)_2_ Laves, AlCr_2_, and r-TiAl_2_ phases. Oxides such as TiO_2_, Al_2_O_3_, Cr_2_O_3_, and a small amount of Y_2_O_3_ were also observed. Owing to the high diffusion rate of Cr, the oxygen diffused faster in Cr_2_O_3_ than Al_2_O_3_, and thus, a fine-grained (Al,Cr)_2_O_3_ was formed after extended oxidation exposure. The Cr_2_O_3_ was then dissolved in Al_2_O_3_ which protected the alloys against oxidation. At 700 °C, γ-TiAl and r-TiAl_2_ phases were found in the interdiffusion region, in addition to Ti(Cr,Al)_2_ Laves phase. More TiO_2_ was formed in the coatings due to the rapid diffusion of oxygen through the coalesced cracks during oxidation, resulting in a higher mass gain. The Ti-5Al-2Sn-2Zr-4Mo-4Cr substrate produced a higher parabolic rate constant and mass gain than the one of Ti-6Al-2Sn-4Zr-2Mo due to the increased oxide growth rate with increasing oxidation temperature. The oxidation kinetics of the Ti-49.4Al-19.5Cr-0.6Y coatings was found to obey a parabolic law at both 600 and 700 °C cyclic oxidation temperatures.

Mitoraj-Królikowska et al. [42] investigated the oxidation kinetics of silicide coated Ti-6Al-1Mn alloys under cyclic oxidation temperatures at 700–800 °C in air. The thickness of the silicide coatings was about 10 μm. The oxidation behaviour of the alloys followed the parabolic law. The estimated activation energy of the silicide coating was 220 kJ/ mol, which was similar to the growth of TiSi phase. The parabolic rate constant of the coated alloys was 2.3 × 10^−14^ g^2^∙cm^−4^∙s^−1^, which is lower than that of the uncoated alloys (1.6 × 10^−11^ g^2^∙cm^−4^∙s^−1^), indicating the effectiveness of silicide coatings in enhancing the oxidation resistance of the Ti-6Al-1Mn alloys. The coated alloys consisted of oxide layers of TiO_2_ on top, a thin intermediate layer of SiO_2_ underneath, with a mixture of TiO_2_ + SiO_2_ at the bottom. The diffusion coefficient of oxygen was lower in SiO_2_ than TiO_2_, and therefore the formation of SiO_2_ was able to effectively hinder the inward diffusion of oxygen. SiO_2_ filled up the voids between TiO_2_ and formed a barrier for further diffusion of oxygen along the grain boundaries. In all, a significant reduction in mass gain was observed in the coated alloys, indicating an increase in oxidation resistance. 

Ti(Al,Si)_3_ coating of about 25 μm in thickness was deposited onto a titanium matrix composite substrate by hot-dip siliconising of the substrate in the Al-10Si melt, followed by annealing at 550 °C for 6 h [43]. The microstructure and oxidation resistance of the coatings were studied under cyclic oxidation at 700–900 °C. The oxidation kinetics of the Ti(Al,Si)_3_ coatings followed the parabolic law for all tested oxidation temperatures. The parabolic rate constant of the Ti(Al,Si)_3_ coatings increased with oxidation temperature. A similar increasing trend in the mass gain of the coatings was also observed. The activation energy of the Ti(Al,Si)_3_ coatings was about 109 kJ/ mol. Upon oxidation, Si atoms dissolved in Ti(Al,Si)_3_ and reacted with the Ti atoms diffused outwardly from the substrate to form Ti_5_Si_4_ silicide at the coating/ substrate interlayer. The Ti_5_Si_4_ silicide continued to grow thicker with increasing oxidation temperature, whereas the Al diffused outwardly to form protective and continuous Al_2_O_3_. The oxide scales comprised of Al_2_O_3_ in the outmost layer and Al_2_O_3_, SiO_2_, and TiO_2_ underneath, which effectively retarded oxygen diffusion. The Ti_5_Si_4_ silicide beneath the oxide scales acted as a barrier, hindering the interfacial reaction between the Ti(Al,Si)_3_ coating and the substrate. Oukati Sadeq et al. [55] reported a similar trend of oxidation behaviour for their Ti-Al-Si coatings due to silicide formation. The oxidised Ti-Al-Si coatings consisted of four oxide layers, with Al_2_O_3_, SiO_2_, and TiO_2_ on top, TiSi_2_ on the second layer, Al_3_Ti on the third layer, and finally the Ti_2_N at the bottom. The presence of silicide TiSi_2_ and Al_3_Ti phases hindered the inward oxygen diffusion and increased high-temperature oxidation resistance. 

**Figure 6:**
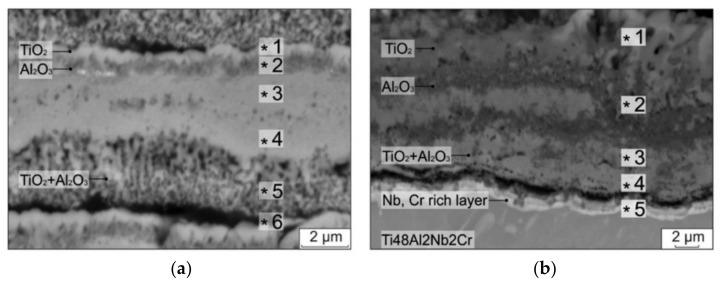
Cross-sectional SEM micrographs of oxide layers formed on (**a**) the Ti alloy substrate and (**b**) Ti-48Al-2Cr-2Nb exposed at 900 °C for 100 h. Reprinted from Surface and Coatings Technology, 319, Iu.N. Maliutina, H. Si-Mohand, J. Sijobert, Ph. Bertrand, D.V. Lazurenko, I.A. Bataev, Structure and oxidation behaviour of γ-TiAl coating produced by laser cladding on titanium alloy, 136-144, Copyright (2017), with permission from Elsevier [44].

Maliutina et al. [44] found that oxidised Ti-48Al-2Cr-2Nb coatings outperformed the base material of Ti-6Al-2Sn-4Zr-2Mo during oxidation at 700–900 °C due to the Cr and Nb additions. The Ti-48Al-2Cr-2Nb coatings exhibited lower mass gain and better oxidation resistance than the Ti-6Al-2Sn-4Zr-2Mo. The effective activation energy of Ti-48Al-2Cr-2Nb coatings and Ti-6Al-2Sn-4Zr-2Mo were 251 and 191 kJ/mol, respectively. The higher effective activation energy of Ti-48Al-2Cr-2Nb coatings corresponds to their lower parabolic rate constant, which resulted in better oxidation resistance than the Ti-6Al-2Sn-4Zr-2Mo. The Nb addition substituted Ti atoms into the crystal structure, which reduced oxygen vacancies and suppressed the growth of TiO_2_. Whereas the Cr addition formed the Ti(Al,Cr)_2_ Laves phases which dissolved in the coating and developed a protective Al_2_O_3_ against further oxidation. The formation of an (Nb, Cr-enriched) layer beneath the oxide layers of TiO_2_, Al_2_O_3_, and TiO_2_ + Al_2_O_3_ was observed (Figure 6). Similar effects of Cr and Nb additions on the oxidation behaviour of Ti-48Al-2Cr-2Nb alloys tested under isothermal conditions from 800–1000 °C [57] is discussed in Section 5.2.2.

Song et al. [45] found that the mass gain of Ta coating on Ti-46.5Al-2.5Cr-1V increased from <2 mg/cm^2^ at 750 °C to <12 mg/cm^2^ at 850 °C. This significant increase in mass gain was due to the inferior high-temperature oxidation resistance of pure Ta. The oxide layers of Ta-coated Ti-46.5Al-2.5Cr-1V consisted of Al_2_O_3_, TiO_2_, and TaO at the beginning of oxidation. With increasing oxidation temperature and time, the TiO_2_ and TaO started to exfoliate whereas the Al_2_O_3_ and Ta_2_O_5_ remain unchanged. At 850 °C, the solid-phase reaction between Al_2_O_3_ and Ta_2_O_5_ formed AlTaO_4_. The formation of Ta_2_O_5_/Al_2_O_3_ and AlTaO_4_/Al_2_O_3_ in Ta-coated Ti-46.5Al-2.5Cr-1V was found effective in inhibiting further diffusion of oxygen, thus increasing oxidation resistance. The beneficial effect of alloy additions in forming a protective oxide against oxidation was also reported by Kim et al. [38], who found that oxidised TiAl-2Nb-2Mo alloys consisted of oxides of TiO_2_/Al_2_O_3_/TiO_2_ + Al_2_O_3_ which grew more thickly at 900 °C than at 800 °C. However, the formation of γ-TiAl and (Nb, Mo-enriched) phases underneath these oxide layers reduced their growth rate.

By optimising the deposition parameter of the laser cladding process, Zambrano Carrullo et al. [46] demonstrated that the oxidation behaviour of Ti-48Al-2Cr-2Nb coatings depended on the thermally formed oxides. All the coatings consisted of γ-TiAl and α_2_-Ti_3_Al phases with different volume fractions. The coatings deposited at low specific energy oxidised at 800 °C and formed a high-volume fraction of γ-TiAl relative to α_2_-Ti_3_Al which favoured the formation of Al_2_O_3_, thus decreasing the growth rate of the oxides. The adherent oxides formed during oxidation were identified as TiO_2_, Al_2_O_3_, TiO_2_ + Al_2_O_3_, and TiN. In contrast, high specific energy coatings consisted of more α_2_-Ti_3_Al phases, which promoted the formation of TiO_2_ due to the depletion of Al. Therefore, the low specific energy coatings had lower mass gain than those deposited at high specific energy and exhibited better oxidation resistance. In contrast, the Ti-6Al-4V substrate had higher mass gain than the Ti-48Al-2Cr-2Nb coatings due to the unsteadiness of the TiO_2_ and Al_2_O_3_ formation.

Hu et al. [39] reported that the phases formed in the as-deposited Ti-59.5Al-13.9Si coatings by self-generated gradient hot-dipping infiltration were in the sequence of Ti(Al,Si)_3_, τ_2_ + L-(Al,Si), and L-(Al,Si) from the substrate. The Ti-59.5Al-13.9Si coatings had lower mass gain than the Ti-6Al-4V substrate. Upon oxidation up to 800 °C, the coatings transformed into new alloy phases consisting of Ti_3_Al, TiAl, and Ti_3_Al + Ti_5_Si_3_. These phases prevented oxygen diffusion into the substrate and thus increased oxidation resistance. Dai et al. [29] reported the similar effect of Ti_5_Si_3_ retarding the inward diffusion of oxygen at a high temperature for Ti-Al-Si coatings. The Ti-Al-Si coatings with a thickness of 400–500 μm were compact and dense, and well-adhered to the substrate. The Ti_5_Si_3_ phase increased with increasing Si content. Furthermore, the addition of Si also refined the oxide grains and promoted the formation of Al_2_O_3_, and thus improving high-temperature oxidation resistance of the coatings. Chen et al. [47] attributed the high oxidation resistance of their Al-Si coatings to the formation of an Al diffused layer, a Ti_5_Si_4_ interlayer, an inner TiAl_3_ layer and an outer Ti-Al-Si alloy layer from the inner substrate to the coating surface. These layers had effectively prevented the diffusion of oxygen at high temperatures. 

Dai et al. [48] investigated the oxidation behaviour of Ti-Al-Nb coatings under isothermal exposure at 800 °C for 1000 h. The maximum thickness of the Ti-Al-Nb coatings was 800–1000 μm. They found that Nb addition in the range of 4.43–9.31 at.%, promoted the formation of Al_2_O_3_ and resulted in lower mass gain which subsequently increased the high-temperature oxidation resistance of the coatings. The substitution of Ti^4+^ with higher valency Nb^5+^ in TiO_2_ lattice reduced the vacancies and diffusion of oxygen, decreasing the oxidation rate. However, adding excess Nb was found to decrease the volume fraction of Al_2_O_3_ relative to TiO_2_, leading to inferior oxidation resistance. This can be explained by the increasing solubility of Al in TiO_2_ with increasing Nb content. 

Dai et al. [49] found that adding various contents of Nb and Si into Ti-Al alloys reduced the mass gain to <2.5 (mg/cm^2^) upon oxidation at 800 °C for 100 h, and promoted the formation of Al_2_O_3_. The parabolic rate constant of the Ti-Al-Nb-Si coatings decreased with increasing Nb and Si content, reaching a minimum of 5.7 × 10^−12^ g^2^∙cm^−4^∙s^−1^ at 9.59 wt.% and 10.52 wt.%, for each respectively. This indicated greater oxidation resistance than other Ti-Al-Nb-Si coatings. Si addition promoted the formation of TiAl_3_ and Ti_5_Si_3_. The increased oxidation resistance was attributed to the formation of SiO_2_ and Al_2_O_3_ on the Ti_5_Si_3_ and TiAl_3_, respectively, which inhibited oxygen diffusion in the coatings. Nb addition provided the same effect of retarding the interdiffusion of oxygen and Ti in the coatings. Therefore, adding Nb and Si simultaneously to Ti-Al alloys has the potential for greater high-temperature oxidation resistance than adding Nb or Si individually.

Tkachenko et al. [56] reported that the oxidation kinetics of Ti-Al-Si based alloys, which were additionally alloyed by Zr and Sn, followed the parabolic law during isothermal oxidation at 700 °C in air. The parabolic rate constant of the reference alloy (Ti-6Al-2Sn-4Zr-2Mo-0.1Si) was higher than Ti-6Al-1.4Si-3Zr, Ti-6Al-1.2Si-2Zr-2Sn and Ti-6Al-1.2Si-2Zr-4Sn alloys. The reduced mass gain for the Ti-Al-Si alloys (compared to the Ti-6Al-2Sn-4Zr-2Mo-0.1Si) was due to the formation of silicide Ti_5_Si_3_ phase, which dissolved in the oxide scales during oxidation, thus inhibiting oxygen diffusion. Furthermore, the addition of Zr in the Ti-Al-Si alloys helped to intensify the precipitation of the silicide and increase its fraction in the Ti matrix. The presence of silicide reduced the porosities and cracks in the oxide layer. The silicide also hindered the recrystallisation of rutile crystals and increased coating/ substrate adherence, resulting in better oxidation resistance than the Zr-free alloys.

#### 5.2.2. Oxidation at 800–1000 °C

As oxidation progresses to a higher temperature, thicker oxide scales and spallation are usually observed in Ti-Al alloys. With the addition of alloying elements, several researchers have demonstrated improved oxidation resistance for alloys at high temperature.

Liu et al. [50] reported that a Mo-alloyed layer with a thickness of about 40 μm was fabricated on the Ti-46.5Al-2.5V-1Cr substrate using plasma surface metallurgy technique. They attributed the increased oxidation resistance of Mo-alloyed Ti-46.5Al-2.5V-1Cr to the formation of Ti_2_AlMo phase, which is impermeable to oxygen. Phases such as Al_2_O_3_, Ti_2_AlMo, and γ-TiAl remained for a prolonged oxidation period, providing further protection to oxidation while reducing the amount of TiO_2_. The effect of Mo addition in improving the oxidation behaviour of γ-TiAl alloys was also reported by Neelam et al. [58]. 

γ-TiAl and Ti(Al,Cr)_2_ Laves phases in Ti-Al-Cr alloys are known to promote the formation of protective Al_2_O_3_. However, as oxidation progressed, the Laves phase in Ti-Al-Cr-Y coatings transformed into Z-phase (Ti_5_Al_3_O_2_), thus losing protection against oxidation due to the presence of the α_2_-Ti_3_Al that developed a fast-growing TiO_2_ [51]. In contrast to Ti-Al-Cr-Y coatings, the Ti-Al-Cr-Zr coatings had a lower oxidation rate and mass gain due to the absence of the deteriorating Ti_5_Al_3_O_2_.

**Figure 7:**
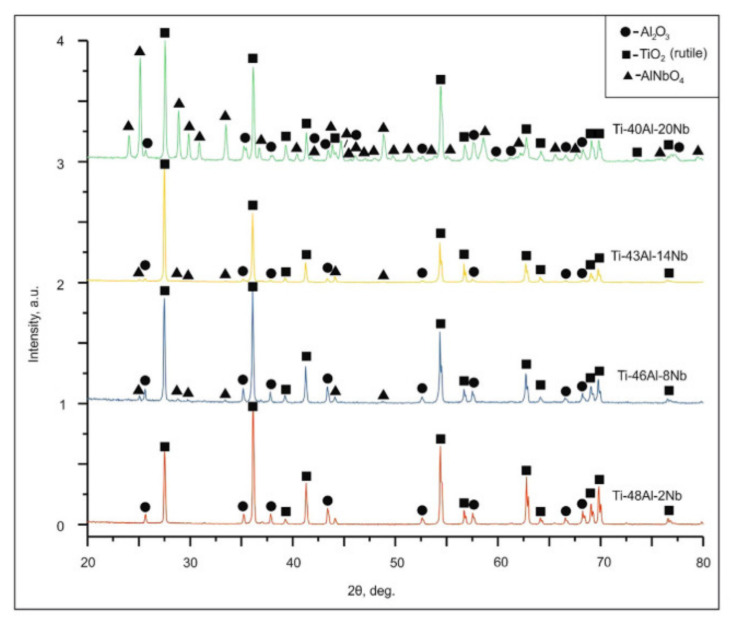
Diffractograms after oxidation at 900 °C. The position of peaks relating to Al_2_O_3_, TiO_2_ (rutile) and AINbO_4_ oxide phases are indicated. Reprinted from Materials Characterization, 163, Daria V. Lazurenko, Ilia S. Laptev, Mikhail G. Golkovsky, Andreas Stark, Jonathan Paul, Ivan Bataev, Alexey A. Ruktuev, Lin Song, Christian Gollwitzer, Florian Pyczak, Influence of the Ti/Al/Nb ratio on the structure and properties on intermetallic layers obtained on titanium by non-vacuum electron beam cladding, 110246, Copyright (2020), with permission from Elsevier [52].

Lazurenko et al. [52] revealed that Al_2_O_3_ and TiO_2_ were predominant in Ti-Al-Nb coatings with Nb content in the range of 5–27 wt.%. These coatings exhibited a low mass gain (<1 mg/cm^2^) after oxidation at 900 °C for 100 h, indicating excellent resistance to oxidation. In contrast, a further increase of Nb content to 34 wt.% increased both the mass gain (to <3 mg/cm^2^) and the oxidation rate due to the formation of AlNbO_4_ (Figure 7), which is highly permeable to oxygen. Without Nb addition, the substrate (Ti-0.086Fe-0.017Cr-0.016Ni-0.012V-0.011C) exhibited a much higher mass gain (<6 mg/cm^2^).

Fröhlich et al. and Zhou et al. [53,54] found that oxidised Ti-Al-Cr coatings consisting of γ-TiAl and Ti(Al,Cr)_2_ Laves phases possessed better oxidation resistance than the Ti-45Al-8Nb and Ti-50Al substrates. The Ti(Al,Cr)_2_ Laves phase promoted the formation of Al_2_O_3_ due to its low oxygen permeability. However, prolonged oxidation exposure decreased the oxidation resistance due to the depletion of Cr whereby the Laves phase dissolved within the alloys and transformed into a single γ-phase and finally into Z-phase which favoured the formation of TiO_2_. Zhou et al. [54] reported that the thickness of both Ti-50Al-15Cr and Ti-50Al-10Cr coatings in their study was about 10 μm. These Ti-Al-Cr coatings had a lower parabolic rate constant at 1000 °C than at 950 °C. They attributed the lower parabolic rate constant at higher oxidation temperature to the diffusivities of oxygen and Al in the Ti-Al-Cr coatings. Oxygen and Al diffused faster in the coatings at 1000 °C than 950 °C, resulting in more rapid formation of Al_2_O_3_. 

Alloying additions in bulk Ti-Al alloys were also effective in improving the oxidation resistance of the alloys. The mass gain and parabolic rate constant of the alloys decreased with alloying additions as shown in Table 3. Lee et al. [57] found that Ti-48Al-2Cr-2Nb alloys exhibited better oxidation resistance than Ti-51Al and Ti-47Al-4Cr under isothermal oxidation at 1000 °C due to the addition of Cr and Nb. The Cr and Nb diffused inwardly and formed a (Cr, Nb-enriched) layer beneath the oxide layers of TiO_2_, Al_2_O_3_, and Al_2_O_3_ + TiO_2_ that retarded the outward diffusion of Ti and inhibited the formation of undesirable TiO_2_. Although several studies have reported the potential of Nb addition for high-temperature oxidation resistance, Shen et al. [60] found that the formation of Nb_2_O_5_ in Nb_2_Al and Nb_3_Al enriched-phases decreased the oxidation resistance of Ti-Al-Nb alloys, particularly with Nb content of more than 20 at.%. The alloys showed spallation during oxidation at 1000 °C. In contrast, the two-phase γ + α_2_ Ti-Al-Nb alloys of about 14.2 at.% Nb exhibited the highest oxidation resistance, consisting primarily of Al_2_O_3_ and a small amount of TiO_2_, giving the lowest mass gain. 

Among all the investigated alloying elements (including Co, Cr, Cu, Fe, Ni, and Mo) added to Ti-15Al-15Si alloys, the addition of Mo produced the highest oxidation resistance with the lowest mass gain (<1 mg/cm^2^) [61]. Mo increased the content of silicide beneath the oxide layers which promoted the formation of Al_2_O_3_. The addition of Sn to Ti-45Al-8.5Nb also showed the same effect of Al_2_O_3_ protection against high-temperature oxidation [59]. The oxide layers consisted of TiO_2_, Al_2_O_3_, TiO_2_ + Al_2_O_3,_ and the (Nb, Sn)-enriched inner layer. The Ti_3_Sn-rich phase promoted the formation of Al_2_O_3_ by inhibiting the interdiffusion between oxygen and Ti. The oxide layers were well adhered to the substrate, thus enhancing spallation resistance. 

In addition to the effect of alloying elements, the content of Al in the Ti-Al alloys also influenced the oxidation of the oxides. Increased Al content usually resulted in a decreased oxidation rate. Al content ranging between 50–63 at.% was found to be beneficial in promoting the protective Al_2_O_3_ for Ti-Al alloys [62]. Furthermore, the formation of Al-rich phases such as TiAl, TiAl_2_, and TiAl_3_ also increased the oxidation resistance of TiAl alloys at high temperature [63,64]. Knaislová et al. [65] reported that the Al content of TiAl and TiAl_3_ phases in their Ti-Al-Si alloys fabricated using the spark plasma sintering (SPS) method were 42 at.% and 55 at.%, respectively. In the present review, most of the Ti-Al alloys showed improvement in oxidation resistance with Al content increasing from 45 to 50 at.%. This could also be attributed to the synergetic effect of the other alloying elements.

In summary, the enhanced high-temperature oxidation resistance for Ti-Al alloys can be attributed to the reduction of the growth rate of oxide scales and the low diffusivity of oxygen at the coating/ substrate interface. 

## 6. The Effect of Alloying Additions on Mechanical Properties 

Alloying modification of Ti-Al alloys can significantly improve oxidation resistance; however, an excess of alloying elements will lead to the degradation of alloy properties due to phase embrittlement. The oxidation reaction is undesirable due to its degradation and consumption of metal. Therefore, a protective layer must be formed to prevent further oxidation, especially under high-temperature conditions. This section discusses the effect of the oxide scale phases on the mechanical properties of the Ti-Al alloys.

### Mechanical Properties

During phase transformation, the grain refinement effect is known to affect the properties of the Ti-Al alloys. For example, the addition of B into γ-TiAl increased the nucleation rate of α-phase in TiAl during the phase transformation from β to α, resulting in grain refinement [66]. According to the Hall-Petch effect [67], grain size decreases with increasing nucleation at the grain boundary, and smaller grain size gives higher hardness. In contrast, a loose morphology reduces the hardness of coatings. Table 4 shows the grain size and mechanical properties of the Ti-Al alloys in terms of microhardness, Young’s modulus, and compressive stress. 

In general, grain growth, and thus grain size, decreased with an increase in Ti_5_Si_3_ silicide. Guan et al. [68] found that Ti-Al-Si alloys with high Si content were able to sustain relatively high resistance to further grain growth when a higher fraction volume of Ti_5_Si_3_ was obtained. The Ti_5_Si_3_ phase was stable and did not grow at higher annealing temperature, thus promoting the formation of smaller grain size. Grain size of less than 100 nm was obtained after annealing at 800 °C. The grain size increased with increasing of annealing temperature. Higher Si content increased the microhardness of Al-Si-Ti alloys due to the formation of Ti_7_Al_5_Si_12_ phase [69]. The microhardness of the alloys increased from 0.5884 to 2.991 GPa by increasing Si content from 3 to 60 wt.%. By keeping Ti content constant, Cabibbo et al. [70] reported that Al and Si content governed the mechanical properties of Ti-Al-Si alloys. A higher ratio of Si/Al content increased the elastic modulus and compression stress of the alloys without affecting their nanoindentation hardness, as shown in Table 4. An increase in the fracture toughness from 0.86 to 1.56 MPa.m^1/2^ for Ti-15Al-15Si and Ti-10Al-20Si alloys was also observed, respectively. The substitution of Al with Si promoted the formation of the strengthening Ti_5_Si_3_ silicide and TiAl phase, which were responsible for improving the mechanical properties of Ti-Al-Si alloys. Similarly, Knaislová et al. [76] found that the microhardness of their Ti-Al-Si alloys increased with increasing Si content due to the strengthening mechanism of slicides such as Ti_5_Si_3_, Ti_5_Si_4_, and TiSi. 

A noteworthy increase in the microhardness of Ti-22Al-24Nb-0.5Mo was attributed to the refinement of Ti_2_AlNb grain size after rolling and heat treatment processes [71]. After hot ring-rolled process, the microhardness of the alloys increased from 2.707 to 3.236 GPa, and grain size decreased from 50 × 10^3^ to 20 × 10^3^ nm. At higher annealing temperatures, lower microhardness was obtained. The compressive stress of the alloys increased from 1295 to 1390 MPa when Nb content in γ-TiAl increased from 3 to 7 at.% [72]. However, no significant change in the microhardness was detected. This could be attributed to the dissolution of Nb into γ-TiAl and α_2_-Ti_3_Al phases, which refined the alloy grains. The increased α_2_-Ti_3_Al fraction relative to γ-TiAl improved the yield strength, whereas high content of Nb in γ-TiAl enhanced the strength and ductility of the alloys. 

Similarly, Yang et al. [73] reported that Fe addition of 0.3–0.5 at.% was able to increase the compressive strength of TiAl-Nb alloys due to grain refinement and solid solution strengthening. A maximum compressive stress of 1869.5 MPa was obtained at 0.3 at.% Fe. The stabilised *B2* phase in Fe retarded the growth of α-phase in TiAl, and thus refined the grain structure. In addition, the solid solution strengthening of the alloys could be attributed to the distortion in γ-phase when the Fe occupied the Al in γ-phase, creating a significant mismatch at the interface. However, compressive stress decreased with a further increase of Fe up to 1.1 at.%. Excessive Fe may have degraded the properties of the alloys by causing segregation of Al in the interdendritic region, leading to coarser grains. Pan et al. [74] presented a similar finding where the compressive strength of TiAl-_x_Sn alloys increased with increasing Sn content but then decreased above a certain level. The compressive strength of the alloys decreased from 3029 to 1501 MPa with increasing Sn content from 1 to 2 and 3 to 5 at.%, respectively. In addition to γ-TiAl and α_2_-Ti_3_Al phases, the brittle phase of Ti_2_Sn started to form when Sn exceeded 3 at.%. This tended to accelerate crack propagation, thus affecting the mechanical properties. 

Mogale et al. [75] reported that the compressive stress of the TiAl alloys increased to 781 MPa with the addition of 1–1.5 at.% Cr but then decreased to 615 MPa at 3 at.% Cr when the alloys were tested at 1000 °C. The addition of Cr of suitable content was able to increase the ductility of the alloys due to their high plasticity behaviour, thus enhancing their compressive properties. He et al. [77] found that the addition of Cr increased the thermal stability and the microhardness of TiAl alloys at an alloying temperature of 1100 °C and discharge pressure of 25 Pa, using a plasma-surface technique. The formation of a diffusion layer on TiAl-Cr alloys prevented the degradation of the substrate and improved the properties of the alloys. Yuan et al. [78] attributed the high resistance to deformation of Ti-Al-Ta alloys to the formation of fine-grained structures around the TaAl_3_ phase due to the interdiffusion between TiAl and Ta addition.

## 7. Conclusions

The oxidation behaviour of complex Ti-Al alloys has been compared by mass gain and parabolic rate constant after 100 h of isothermal exposure (or equivalent to 300 cycles under cyclic exposure). The phase structures and oxidation kinetics of the indicated alloys were reviewed at their respective oxidation temperature ranges: namely, 600–800 °C and 800–1000 °C. The effect of alloying elements on the mechanical properties of Ti-Al alloys was also discussed. The following conclusions can be drawn from the preceding review:

- The oxidation behaviour of complex Ti-Al alloys closely follows parabolic kinetics at 600–1000 °C. The main oxidation products of these alloys are TiO_2_, Al_2_O_3_, TiO_2_ + Al_2_O_3_, and alloy enriched oxides such as Cr_2_O_3_, SiO_2_, Ta_2_O_5_, and AlTaO_4_. These alloy enriched oxides have been found to be beneficial in decreasing oxygen solubility and diffusivity, hence promoting the formation of protective Al_2_O_3_.
- The inclusion of alloying elements can improve the oxidation protection of Ti-Al alloys at high temperature by forming an oxygen-diffusion barrier upon oxidation that suppresses the interdiffusion between the oxygen and metal ions. Among all the investigated alloys, Si appears to be the most beneficial alloying addition, achieving excellent oxidation resistance at high temperatures. Si addition of about 5–13 at.% forms titanium silicide that promotes the formation of protective Al_2_O_3_ and inhibits the growth of TiO_2_. This strengthening silicide also improves the mechanical properties of Ti-Al alloys.
- The enhancement of hardness can be attributed to the formation of new phases, reduction in the grain size, and solid solution strengthening by element additions to Ti-Al alloys.

Adding alloying elements to Ti-Al alloys has significantly improved their oxidation resistance and mechanical performance, specifically at high temperatures. The Ti-Al alloys without element addition exhibit increases in mass gain and parabolic rate constant with increasing oxidation temperature due to the high oxygen diffusion in Ti. This leads to the formation of porous TiO_2_, which usually cracks and degrades the oxidation resistance of the alloys. In contrast, alloying elements dissolve in the oxide layer of the Ti-Al alloys and decrease the oxygen diffusion in Ti, resulting in the formation of a more compact Al_2_O_3_ with increasing oxidation temperature. The alloying additions have been found to reduce the oxidation rate for up to 1000 °C with a lower mass gain and parabolic rate constant compared to Ti-Al alloys. The content of the respective alloying elements governs the thermodynamic stability of TiO_2_ and Al_2_O_3_ of the Ti-Al alloys. Therefore, understanding the behaviour of oxide scales in the oxidation process helps to distinguish the different formation mechanisms of the fast-growing TiO_2_, compact Al_2_O_3_, and alloy enriched oxides. Ultimately, the synergistic effect of the different alloying elements to form tribo-oxides may provoke interest in further exploring the tribological performance of these alloys. 

## Figures and Tables

**Table 1:** Pilling-Bedworth ratios (R_PB_) of metallic oxides.

Oxide	Formula	Pilling-Bedworth Ratio (R_PB_)	Reference
Potassium oxide	K_2_O	0.45	[13]
Calcium oxide	CaO	0.64	[17]
Barium oxide	BaO	0.67	[17]
Magnesium oxide	MgO	0.81	[17]
Sodium oxide	Na_2_O	0.97	[13]
Aluminium oxide	Al_2_O_3_	1.28	[17]
Lead (II) oxide	PbO	1.28	[17]
Zirconium (IV) oxide	ZrO_2_	1.56	[17]
Zinc oxide	ZnO	1.58	[17]
Nickel (II) oxide	NiO	1.65	[17]
Iron (II) oxide	FeO	1.70	[18]
Copper (II) oxide	CuO	1.70	[18]
Titanium (IV) oxide	TiO_2_	1.78	[13]
Manganese (II) oxide	MnO	1.80	[18]
Chromium (III) oxide	Cr_2_O_3_	2.07	[17]
Iron (III) oxide	Fe_2_O_3_	2.14	[17]
Silicon dioxide	SiO_2_	2.15	[17]
Tantalum (V) oxide	Ta_2_O_5_	2.47	[17]
Niobium (V) oxide	Nb_2_O_5_	2.69	[17]
Vanadium (V) oxide	V_2_O_5_	3.25	[17]
Molybdenum (VI) oxide	MoO_3_	3.30	[17]
Tungsten (VI) oxide	WO_3_	3.30	[17]

**Table 2:** Summary of the high-temperature exposure, nominal composition, mass gain, and parabolic rate constant for Ti-Al alloy coatings and their uncoated substrates developed by various deposition techniques.

Exposure	Nominal Composition of Coating (wt.%)	Nominal Composition of the Substrate (wt.%)	Mass Gain of Coating (mg/cm^2^)	Mass Gain of the Substrate (mg/cm^2^)	Parabolic Rate Constant of Coating (g^2^∙cm^−4^∙s^−1^)	Parabolic Rate Constant of the Substrate (g^2^∙cm^−4^∙s^−1^)	Deposition Technique	Reference
600 °C; C	Ti-49.4Al-19.5Cr-0.6Y *	Ti-5Al-2Sn-2Zr-4Mo-4Cr	<0.10	<0.30		1.3 × 10^−13^	Magnetron sputtering	[41]
	Ti-49.4Al-19.5Cr-0.6Y *	Ti-6Al-2Sn-4Zr-2Mo	<0.15	<0.25		6.2 × 10^−14^		
700 °C; C	Ti-49.4Al-19.5Cr-0.6Y *	Ti-5Al-2Sn-2Zr-4Mo-4Cr	<0.25	<2.25		4.9 × 10^−12^		
	Ti-49.4Al-19.5Cr-0.6Y *	Ti-6Al-2Sn-4Zr-2Mo	<0.25	<1		9.1 × 10^−13^		
700 °C; C	TiSi_2_ (Silicide)	Ti-6Al-1Mn *	<0.1	<5	2.3 × 10^−14^	1.6 × 10^−11^	Pack cementation	[42]
700 °C; C	Ti-61Al-14Si *	TiB whiskers reinforced Ti-6Al-4V (TiBw/Ti-6Al-4V)	<0.36	<4.01	3.6 × 10^−12^		Hot-dip siliconising	[43]
800 °C; C	Ti-61Al-14Si *	TiBw/Ti-6Al-4V	<1.33	<22.18	4.9 × 10^−11^			
900 °C; C	Ti-61Al-14Si *	TiBw/Ti-6Al-4V	<3.98	<54.21	1.1 × 10^−10^			
700 °C; I	Ti-48Al-2Cr-2Nb	Ti-6Al-2Sn-4Zr-2Mo	<0.3	<0.3			Laser cladding	[44]
800 °C; I	Ti-48Al-2Cr-2Nb	Ti-6Al-2Sn-4Zr-2Mo	<0.8	<4.8				
900 °C; I	Ti-48Al-2Cr-2Nb	Ti-6Al-2Sn-4Zr-2Mo	<3.5	<31.4				
750 °C; I	Ta	Ti-46.5Al-2.5Cr-1V	<2	<4			Double glow plasma surface alloying treatment	[45]
850 °C; I	Ta	Ti-46.5Al-2.5Cr-1V	<12	<28				
800 °C; I	Ti-48Al-2Cr-2Nb	Ti-6Al-4V	<2.5	<20			Laser cladding	[46]
800 °C; I	Ti-41.6Al-4.8Si	Ti-6Al-4V	<0.25	<5			Laser surface alloying	[29]
	Ti-38.1Al-9.5Si		<0.15					
	Ti-27.5Al-10.3Si		<0.27					
800 °C; I	Ti-59.5Al-13.9Si	Ti-6Al-4V	<5	<45			Self-generated gradient hot-dipping infiltration	[39]
850 °C; C	Al-Si	Ti-6Al-4V	<2	<8			Mechanical alloying	[47]
800 °C; I	Ti-54.67Al-4.43Nb *	Ti-6Al-4V	<6	<5			Laser surface alloying	[48]
	Ti-50.88Al-5.61Nb *		<5.5					
	Ti-48.11Al-7.08Nb *		<5					
	Ti-44.65Al-9.31Nb *		<4					
	Ti-40.15Al-12.34Nb *		<8					
800 °C; I	Ti-Al-5.46Nb-5.30Si	Ti-6Al-4V	<2.5	<5	2.8 × 10^−11^		Laser surface alloying	[49]
	Ti-Al-10.84Nb-5.79Si		<2		1.5 × 10^−11^			
	Ti-Al-16.47Nb-5.93Si		<2		1.4 × 10^−11^			
	Ti-Al-5.46Nb-11.01Si		<2		1.1 × 10^−11^			
	Ti-Al-9.59Nb-10.52Si		<1.5		5.7 × 10^−12^			
850 °C; C	Mo	Ti-46.5Al-2.5V-1Cr *	<3	<14			Plasma surface metallurgy	[50]
850 °C; C	Ti-46Al-36Cr-4Zr *	Ti-48Al-2Cr-2Nb *	<0.125	<1.75			Magnetron sputtering	[51]
	Ti-58Al-14Cr-1Y *	Ti-48Al-2Cr-2Nb *	<0.25	<1.75				
	Ti-46Al-39Cr-4Zr *	Ti-43.5Al-4Nb-1Mo-0.1B *	<0.125	<1.50				
	Ti-59Al-14Cr-2Y *	Ti-43.5Al-4Nb-1Mo-0.1B *	<0.25	<1.50				
900 °C; C	Ti-46Al-36Cr-4Zr *	Ti-48Al-2Cr-2Nb *	<0.25	<1.25				
	Ti-58Al-14Cr-1Y *	Ti-48Al-2Cr-2Nb *	<0.5	<1.25				
	Ti-46Al-39Cr-4Zr *	Ti-43.5Al-4Nb-1Mo-0.1B *	<0.5	<1.50				
	Ti-59Al-14Cr-2Y *	Ti-43.5Al-4Nb-1Mo-0.1B *	<0.5	<1.50				
950 °C; C	Ti-46Al-36Cr-4Zr *	Ti-48Al-2Cr-2Nb *	<0.25	-				
	Ti-58Al-14Cr-1Y *	Ti-48Al-2Cr-2Nb *	<0.625	-				
	Ti-46Al-39Cr-4Zr *	Ti-43.5Al-4Nb-1Mo-0.1B *	<0.125	-				
	Ti-59Al-14Cr-2Y *	Ti-43.5Al-4Nb-1Mo-0.1B *	<0.25	-				
900 °C; C	Ti-33Al-5Nb	Ti-0.086Fe-0.017Cr-0.016Ni-0.012V-0.011C	<0.9	<6			Non-vacuum electron beam cladding	[52]
	Ti-28Al-17Nb		<0.5					
	Ti-24Al-27Nb		<1					
	Ti-20Al-34Nb		<3					
900 °C; C	Ti-51Al-12Cr *	Ti-45Al-8Nb *	<1.1	<1.5			Magnetron sputtering	[53]
950 °C; C	Ti-51Al-12Cr *	Ti-45Al-8Nb *	<1.2	-				
950 °C; I	Ti-50Al-10Cr *	Ti-50Al *	<3.8	-	3.2 × 10^−11^		Magnetron sputtering	[54]
	Ti-50Al-15Cr *		<0.9		1.1 × 10^−12^			
1000 °C; I	Ti-50Al-10Cr *		<1.5		3.6 × 10^−12^			
	Ti-50Al-15Cr *		<0.6		8.3 × 10^−13^			
950 °C; C	Ti-50Al-10Cr *		<1.2					
	Ti-50Al-15Cr *		<0.4					
1000 °C; C	Ti-50Al-10Cr *		<2					
	Ti-50Al-15Cr *		<0.5					
1000 °C; I	Ti-Al-Si	Ti-6Al-4V	<0.5	<3.5			Hot-dip siliconising	[55]

I: isothermal, C: cyclic oxidation exposure. *: nominal composition in at.%.

**Table 3:** Summary of the high-temperature exposure, nominal composition, mass gain, and parabolic rate constant for bulk Ti-Al alloy prepared by different methods.

Exposure	Nominal Composition (wt.%)	Mass Gain (mg/cm^2^)	Parabolic Rate Constant (g^2^∙cm^−4^∙s^−1^)	Preparation Method	Reference
700 °C; I	Ti-6Al-2Sn-4Zr-2Mo-0.1Si	<1	2.14 × 10^−12^	Argon arc melting	[56]
	Ti-6Al-1.4Si	<0.50	8.29 × 10^−13^		
	Ti-6Al-1.4Si-3Zr	<0.40	4.53 × 10^−13^		
	Ti-6Al-1.2Si-2Zr-2Sn	<0.40	5.53 × 10^−13^		
	Ti-6Al-1.2Si-2Zr-4Sn	<0.45	5.84 × 10^−13^		
800 °C; I	Ti-51Al	<0.5		Vacuum arc melting and induction skull melting	[57]
	Ti-47Al-4Cr	<0.8			
	Ti-48Al-2Cr-2Nb	<0.7			
900 °C; I	Ti-51Al	<1.7			
	Ti-47Al-4Cr	<9.5			
	Ti-48Al-2Cr-2Nb	<2			
1000 °C; I	Ti-51Al	<10			
	Ti-47Al-4Cr	<27.5			
	Ti-48Al-2Cr-2Nb	<2.5			
850 °C; I	Ti-46.5Al-3.5Nb-2Cr-0.3B	<1.75		Vacuum arc melting	[58]
	Ti-46.5Al-3.5Nb-1Cr-1Mo-0.3B	<1.5			
	Ti-46.5Al-3.5Nb-2Mo-0.3B	<1.25			
1000 °C; I	Ti-45Al-8.5Nb-Sn *	<4.0		Simple press and sinter route	[59]
	Ti-45Al-8.5Nb-3Sn *	<3.2			
1000 °C; I	Ti-47.5Al-5Nb *	<5		Arc-melting	[60]
	Ti-42.8Al-14.2Nb *	<2			
	Ti-40Al-20Nb *	<4			
	Ti-30Al-40Nb *	<8			
1000 °C; I	Ti-15Al-15Si	<20		Self-propagating high- temperature synthesis	[61]
	Ti-15Al-15Si-15Co	<18			
	Ti-15Al-15Si-15Cr	<5			
	Ti-15Al-15Si-15Cu	<20			
	Ti-15Al-15Si-15Fe	<18			
	Ti-15Al-15Si-15Mo	<1			
	Ti-15Al-15Si-15Ni	<18			

I: isothermal, C: cyclic oxidation exposure. *: nominal composition in at.%.

**Table 4:** Grain size and mechanical properties of the Ti-Al alloys.

Nominal Composition (wt.%)	Grain Size (nm)	Microhardness (GPa)	Young’s Modulus (GPa)	Compressive Stress (MPa)	Reference
Annealed at 800 °C					[68]
Ti-28.2Al-18.5Si	25				
Ti-40Al-9.5Si	40				
Ti-44.7Al-5.6Si	55				
Ti-23.5Al-6.5Si	56				
Ti-38.4Al-5Si	50				
Ti-45.6Al-1.2Si	35				
Ti-28Al-7Si	95				
Ti-28Al-10Si	50				
Ti-20Al-13.5Si	53				
Ti-20Al-15Si	40				
Ti-52Al-2.5Si	56				
Annealed at 900 °C					
Ti-28.2Al-18.5Si	72				
Ti-40Al-9.5Si	96				
Ti-44.7Al-5.6Si	120				
Ti-23.5Al-6.5Si	168				
Ti-38.4Al-5Si	96				
Ti-45.6Al-1.2Si	64				
Ti-28Al-7Si	160				
Ti-28Al-10Si	104				
Ti-20Al-13.5Si	96				
Ti-20Al-15Si	94				
Ti-52Al-2.5Si	144				
Annealed at 1100 °C					
Ti-28.2Al-18.5Si	160				
Ti-40Al-9.5Si	220				
Ti-44.7Al-5.6Si	170				
Ti-23.5Al-6.5Si	340				
Ti-38.4Al-5Si	190				
Ti-45.6Al-1.2Si	220				
Ti-28Al-7Si	180				
Ti-28Al-10Si	240				
Ti-20Al-13.5Si	200				
Ti-20Al-15Si	160				
Ti-52Al-2.5Si	280				
Al-3Ti-2Ti		0.5884			[69]
Al-6Si-2Ti		0.6375			
Al-10Si-2Ti		0.6865			
Al-12Si-2Ti		0.9317			
Al-14Si-2Ti		1.324			
Al-18Si-2Ti		1.618			
Al-30Si-2Ti		2.059			
Al-60Si-2Ti		2.991			
Ti-10Al-20Si	d_TiAl_ = 950	1.02	57	340	[70]
	d_Ti5Si3_ = 160				
Ti-15Al-15Si	d_TiAl_ = 320	1.0	33	330	
	d_Ti5Si3_ = 140				
Ti-22Al-24Nb-0.5Mo *	50 × 10^3^	2.707			[71]
After rolling	20–50 × 10^3^	3.236			
Heat treated 980 °C; aged 830 °C	10–40 × 10^3^	3.158			
Heat treated 980 °C; aged 800 °C	10–40 × 10^3^	3.354			
Heat treated 960 °C; aged 830 °C	10–40 × 10^3^	3.138			
Heat treated 960 °C; aged 800 °C	10–40 × 10^3^	3.315			
Ti-48Al-2Mn-3Nb *		19.37		1295	[72]
Ti-48Al-2Mn-4Nb *		19.53		1320	
Ti-48Al-2Mn-5Nb *		19.09		1340	
Ti-48Al-2Mn-6Nb *		19.24		1360	
Ti-48Al-2Mn-7Nb *		19.58		1390	
Ti-46Al-5Nb-0.1B *				1750	[73]
Ti-46Al-5Nb-0.1B-0.3Fe *				1869.5	
Ti-46Al-5Nb-0.1B-0.5Fe *				1830	
Ti-46Al-5Nb-0.1B-0.7Fe *				1710	
Ti-46Al-5Nb-0.1B-0.9Fe *				1520	
Ti-46Al-5Nb-0.1B-1.1Fe *				1450	
TiAl *				2870	[74]
TiAl-1Sn *				3029	
TiAl-2Sn *				2960	
TiAl-3Sn *				2634	
TiAl-5Sn *				1501	
Ti-47.5Al-1Cr *				700	[75]
Ti-47.5Al-1.5Cr *				781	
Ti-47.5Al-3Cr *				615	

*: nominal composition in at.%

## Data Availability

Data sharing not applicable.
